# Immunogenicity of parenterally delivered plant-derived small and medium surface antigens of hepatitis B virus

**DOI:** 10.1007/s00299-016-1944-8

**Published:** 2016-02-23

**Authors:** Olga Fedorowicz-Strońska, Józef Kapusta, Marcin Czyż, Małgorzata Kaczmarek, Tomasz Pniewski

**Affiliations:** Institute of Plant Genetics, Polish Academy of Sciences, Strzeszyńska 34, 60-479 Poznan, Poland; Institute of Biotechnology and Antibiotics, Starościńska 5, 02-516 Warsaw, Poland

**Keywords:** Anti-HBs antibodies, Anti-preS2 antibodies, IgG isotypes, M-HBsAg, S-HBsAg, Plant-derived vaccines

## Abstract

*****Key message***:**

**Intramuscularly delivered plant-derived M-HBsAg was compared to S-HBsAg, and as a result elicited specific anti-preS2 antibodies and significantly higher titre of anti-HBs antibodies, together with IgG isotype profile indicating some Th1 polarisation, apart from the main Th2 response.**

**Abstract:**

HBV prevalence is still threatening, regardless of prevention programmes using vaccines containing S-HBsAg, supplemented by third-generation vaccines, comprising also M- and L-HBsAg. Plant expression systems offer a cost-effective production option of the antigens. Plant-derived S- and M-HBsAg, intramuscularly delivered to mice, elicited anti-HBs antibodies several times higher than high responsiveness threshold titre. M-HBsAg induced stronger response of anti-HBs and also specific anti-preS2 antibodies. IgG isotype profiles indicated mainly Th2 response, yet Th1 polarisation was also pointed out, in some larger extent for M-HBsAg. These results correspond to research on CHO-derived M-HBsAg vs. commercial vaccines based on S-HBsAg and support potency of plant-derived antigens as alternative injection vaccines.

Hepatitis B virus (HBV) is the causative agent of one of the most common and dangerous human viral diseases. Although efficient subunit vaccines based on the small surface antigen of the virus, S-HBsAg, were implemented into vaccination programmes 30 years ago, it is estimated that one-third of the global population has been infected with HBV, the number of chronic carriers exceeds 370 million and about 800,000 people die every year due to the post-disease complications, mostly in developing countries. Therefore, a lot of research effort has been devoted to create both more efficacious and economically feasible vaccines. This resulted in the development of specimens which contain, in addition to S-HBsAg, other HBV surface antigens displaying immunogenic domains: medium M-HBsAg with preS2 and/or large L-HBsAg with preS1 and preS2 (Shouval et al. 2015), as well as attempts to produce these antigens in plant expression systems. Numerous research demonstrated that plants can be employed as efficient bioreactors producing various native antigens capable of inducing efficacious immune response, equivalent to conventional specimens. Among the most advanced plant-derived biopharmaceuticals, glucocerebrosidase for the treatment of Gaucher disease produced in carrot cells (Protalix™) has been already approved by FDA, while quadrivalent influenza vaccine produced by Medicago Inc. in *Nicotiana benthamiana* via viral vector-mediated expression, has been enrolled in phase II of clinical trials (Ward et al. 2014). Also HBV antigens, mostly S-HBsAg, were effectively produced in plants and their immunogenicity was proved, both after oral or parenteral administration (Pniewski 2014). Yet in those studies, almost exclusively the titre of anti-HBs antibodies as the most general response to HBV antigens, was analysed. Other components of immune response were not thoroughly investigated, while these determine response type, vital for the efficacy of a potential vaccine. Moreover, details of the reaction to parenterally delivered purified plant-derived HBV antigens require extensive studies as they can play irreplaceable role in mixed immunisation schemes comprising injection priming and oral boost with cell-encapsulated antigens.

The aim of this study was to assess the effectiveness of immunisation using plant-derived S- and M-HBsAg, administrated parenterally to mice. Total anti-HBs and anti-preS2 antibodies were assayed to estimate specific immunogenicity of antigens, while analysis of anti-HBs IgG subclass distribution was carried out to determine the pattern of the immune response.

The HBV antigens were derived from previously obtained transgenic tobacco plants producing ca. 10 µg/g FW of VLP-assembled S- or M-HBsAg, as assayed by ELISA tests based on mAbs specific to ‘a’ epitope and using S-HBsAg (Meridian Life Science) or CHO-expressed M/L-HBsAg (kindly provided by Prof. Reinhold Schirmbeck, University of Ulm, Germany) as standards. The plant-produced antigens were purified from leaf extracts (PBS pH 7.4 with 1 % v/v Tween^®^ 20, ratio 1:5) by ultracentrifugation (60,000 rpm, 20 h, 15 °C) in CsCl step gradients (10 ml composed of four equal parts of solutions 1.1–1.4 g/ml). The VLP-assembled antigens located mainly in 8th–9th of 1 ml fractions, corresponding to approx. 1.2 g/ml of CsCl density. After dialysis, caesium concentration dropped to 0.7 µg/ml as assayed by ICP MS. Final content of S- and M-HBsAg amounted 1.693 and 0.727 µg/ml, respectively, in comparison to 0.1–0.7 µg/ml achieved previously using sucrose gradient (Pniewski 2014), and then was approximately fivefold concentrated using Amicon^®^ Ultra filtration columns (Millipore).

Preparations containing 0.3 (priming) or 0.1 µg (boosting) of S- and M-HBsAg VLPs or equivalent volume of control preparation, were adjuvanted with 10 % v/v alhydrogel (Sigma) in total volume of 100 µl PBS and administered to mice (5 per group) by intramuscular injection on days 0 and 28. Total anti-HBs antibodies in mice sera were assayed three times using analytical kit Monolisa™ Anti-HBs PLUS Assay (BioRad). IgG isotypes were analysed by in-house ELISA tests using S-HBsAg and appropriate IgG1, IgG2a and IgG2b anti-HBs mAbs (Meridian Life Science) as standards and then HRP-conjugated goat polyclonal antibodies, specific to particular Ig isotypes (Rockland). Anti-preS2 antibodies in sera were assayed by the in-house sandwich ELISA test, using anti-preS2 mAb (Meridian Life Science), 1–25 aa preS2 fragment (American Peptide) and goat anti-mouse IgGAM (Sigma) followed by anti-goat HRP-conjugated polyclonal rabbit antibody (Sigma).

Parenterally administered plant-derived HBs antigens elicited significant immune responses while no reaction was observed in control mice. M-HBsAg appeared more immunogenic than S-HBsAg as anti-HBs antibody titres were significantly higher at all time points after boosting to reach finally 1165 mIU/ml, in comparison to 765 mIU/ml (Fig. 1a). Among anti-HBs IgG subclasses, IgG1 was the predominant for both antigens. Although final IgG1 concentrations did not differ significantly between groups, it can be observed that M-HBsAg evoked stronger IgG1 response (28,000 vs. 24,000 ng/ml, respectively), which continued to grow, while the response to S-HBsAg appeared to slow down (Fig. 1b). The IgG2a and IgG2b isotypes could be detected as late as 15 weeks after boosting and these responses were significantly lower than for IgG1 (not shown). Particular antigens induced distinctly differed response patterns (Figs. 1c, d). S-HBsAg in comparison to M-HBsAg induced significantly higher IgG2a, but almost equally with IgG2b (concentration ratio 1.1). For M-HBsAg, production of IgG2a vs. IgG2b, although statistically homogenous (not shown), was visibly lower (ratio 0.4). A similar tendency was observed when concentration of those IgG subclasses was collated to IgG1. The ratios of IgG1/IgG2a and IgG1/IgG2b for S-HBsAg amounted 31 and 38 respectively, while conversely for M-HBsAg these were 70 and 31, reflecting more intense production of IgG2b antibodies. Humoral response against preS2 domain was observed only in mice delivered M-HBsAg, with titres ranging from 1:100 to 1:400, while all mice immunised with S-HBsAg were negative (Fig. 1e).

**Fig. 1:**
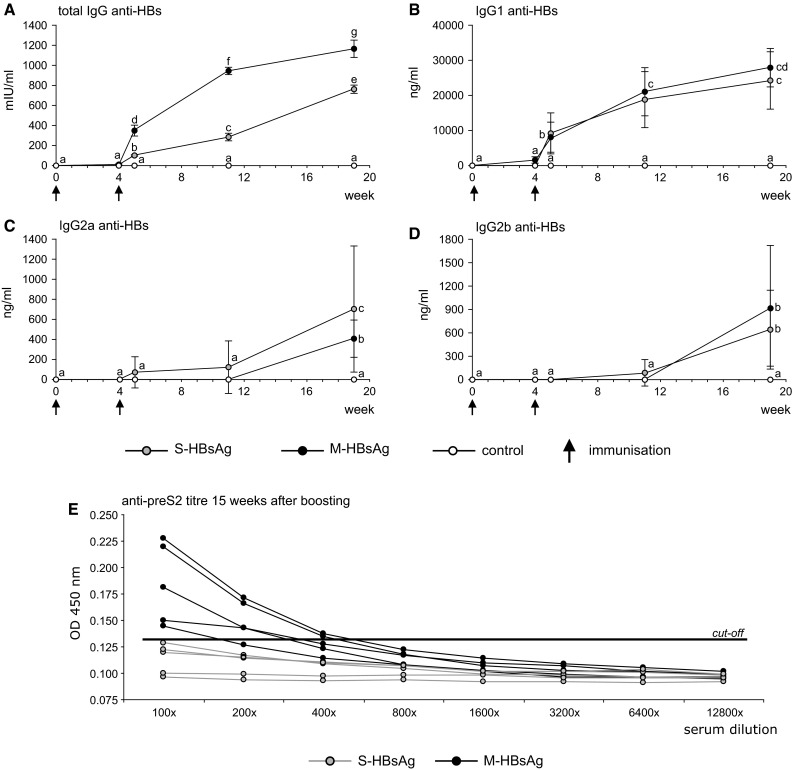
Humoral response of anti-HBs (**a**–**d**) and anti-preS2 (**e**) antibodies, elicited in mice sera after intramuscular injection of plant-derived S-HBsAg (*grey circles*) or M-HBsAg (*black circles*) and control extract from wild-type tobacco (*white circles*). Mice were immunised (*arrows*) with equivalent antigen doses at day 0 (priming—0.3 μg) and after 4 weeks (boosting—0.1 μg). Anti-HBs antibodies: **a** total Ig (mIU/ml), **b** IgG1 (ng/ml), **c** IgG2a (ng/ml), **d** IgG2b (ng/ml); comparisons of responses made separately for total Ig and IgG subclasses, using ANOVA for repeated measures with Duncan’s post hoc test (Statistica 8.0), letter indexes indicate homogenous groups at *p* = 0.05; antibody levels calculated for each group as arithmetic means (*n* = 5) with standard deviations (SD) from three assays on the base of the standard curve for total Ig or each IgG subclass. Anti-preS2 antibodies (**e**) assayed 15 weeks post boosting and expressed as the titre, calculated by comparison of a serum dilution to the cut-off value—mean read-outs (absorbance at 450 nm wavelength) plus three standard deviations of control mice; note: data for dilutions from the range 25,600–102,400 not shown, due to OD_450_ values identical with these for dilution 12,800

In this study, we characterised basic properties of the immune response induced by plant-derived S- and M-HBsAg delivered intramuscularly. These elicited anti-HBs antibodies several times higher than titre considered as high responsiveness threshold (100 mIU/ml) and comparably to commercial vaccines, e.g. Engerix B^®^ administered to mice in dose 0.5–2.0 µg. Here, the responses were induced by several times lower antigen doses (0.1–0.3 µg) and without special enforcement (e.g. Freund’s adjuvant or boosting with a commercial vaccine), but they gave results comparable or higher than reported previously (see Pniewski 2014 for review). We also showed—for the first time—that parenterally delivered plant-derived M-HBsAg triggered also specific anti-preS2 antibodies. The preS2 domain, apart from its innate immunogenicity, probably enhanced the reaction triggered by S domain alone. M-HBsAg induced total anti-HBs antibodies more efficiently than S-HBsAg, similarly to commercial vaccines, e.g. Bio-Hep-B™. Moreover, as IgG isotypes reflect Th1 and Th2 immune responses, the results show that although Th2 response was the main component of the immune response for both plant-derived antigens, M-HBsAg was able to induce the Th1 polarisation to a slightly larger extent.

Plant-produced S- and M-HBsAg require further studies on purification and plant-pattern glycosylation, due to its possible impact on immunogenicity (Ward et al. 2014). Also other details of elicited immune response, e.g. changes in lymphocyte population, should be investigated. However, our findings correspond to observations previously reported for CHO-derived vaccines containing M-HBsAg and compared to those based on S-HBsAg (Madaliński et al. 2002, Krawczyk et al. 2014, Shouval et al. 2015). Hence the obtained results support potency of purified plant-derived S- and M-HBsAg as self-contained injection vaccines or used in mixed parenteral-oral immunisation schemes.

## **Author contribution statement**

OFS, JK and TP conceived and designed the study. OFS performed most of all experiments with the help of JK—mouse immunisation trials, MC—antibody analysis, and TP—plant material generation. MK and TP performed statistical analysis. OFS, MC and TP wrote the paper.
